# Contact splitting in dry adhesion and friction: reducing the influence of roughness

**DOI:** 10.3762/bjnano.10.1

**Published:** 2019-01-02

**Authors:** Jae-Kang Kim, Michael Varenberg

**Affiliations:** 1 George W. Woodruff School of Mechanical Engineering, Georgia Institute of Technology, Atlanta, GA 30332, USA

**Keywords:** biomimetics, contact splitting, gecko adhesion, surfaces, tribology

## Abstract

Splitting a large contact area into finer, sub-contact areas is thought to result in higher adaptability to rough surfaces, stronger adhesion, and a more uniform stress distribution with higher tolerance to defects. However, while it is widely believed that contact splitting helps to mitigate the negative effects of roughness on adhesion- and friction-based attachment, no decisive experimental validation of this hypothesis has been performed so far for thin-film-based adhesives. To this end, we report on the behavior of original and split, wall-shaped adhesive microstructures on different surfaces ranging across four orders of magnitude in roughness. Our results clearly demonstrate that the adhesion- and friction-driven attachment of the wall-shaped microstructure degrades, regardless of the surface waviness, when the surface roughness increases. Second, splitting the wall-shaped microstructure indeed helps to mitigate the negative effect of the increasing surface unevenness by allowing the split microstructure to adapt more easily to the surface waviness and by reducing the effective average peeling angle. These findings can be used to guide the development of biomimetic shear-actuated adhesives suitable for operation not only on smooth but also on rough surfaces.

## Introduction

Biological attachment systems based on thin-film adhesion have drawn significant interest during the last two decades because of their ability to operate on nearly any surface, their efficient control of detachment and their high resistance to contamination [1–4]. However, mimicking their micro- and nanoscale architecture still presents a manufacturing challenge due to their complex hierarchical geometry [3,5–6]. Instead, much simpler structures in the shape of mushrooms, wedges and flaps have been introduced to replicate the operation of biological thin-film-based contact elements [7–14]. These artificial structures perform reasonably well on smooth substrates, which makes them suitable for industrial applications such as silicon wafer or display panel handling [15–17].

Because the ultimate goal of mimicking biological adhesives is to achieve efficient and easily controllable adhesion on any surface, it is important to understand how to overcome the negative effects of roughness on attachment of thin films. It was shown that gecko adhesion is lower if the substrate waviness wavelength is comparable to the lamella length (low-level-hierarchy attachment element) and inter-lamella spacing, and if the substrate roughness is comparable to the lateral dimension of a single spatula (high-level-hierarchy attachment element) [18–19]. Several studies performed with artificial fibrillar structures reported that their attachment abilities are reduced if the fibril dimensions are similar to the root-mean-square roughness, the mean spacing between local peaks, and the surface waviness characteristics of the substrate [20–23]. Analogous negative effects of roughness on adhesion and friction of biomimetic thin-film based structures were also recently demonstrated [24–28], although positive effects associated with an increase in roughness were reported as well [29].

In general, splitting a large contact area into finer sub-contact areas is thought to result in higher adaptability to rough surfaces, stronger adhesion, and more uniform stress distribution with higher tolerance to defects [30–35]. However, although it is generally believed that contact splitting helps to mitigate the negative effects of roughness on adhesion- and friction-based attachment [23,30,32], no decisive experimental validation of this hypothesis has been performed so far for thin-film-based adhesives. To this end, here we report the adhesive and frictional behavior of original and carefully split wall-shaped adhesive microstructures [36] on different surfaces ranging across four orders of magnitude in roughness.

## Results and Discussion

**Figure 1:**
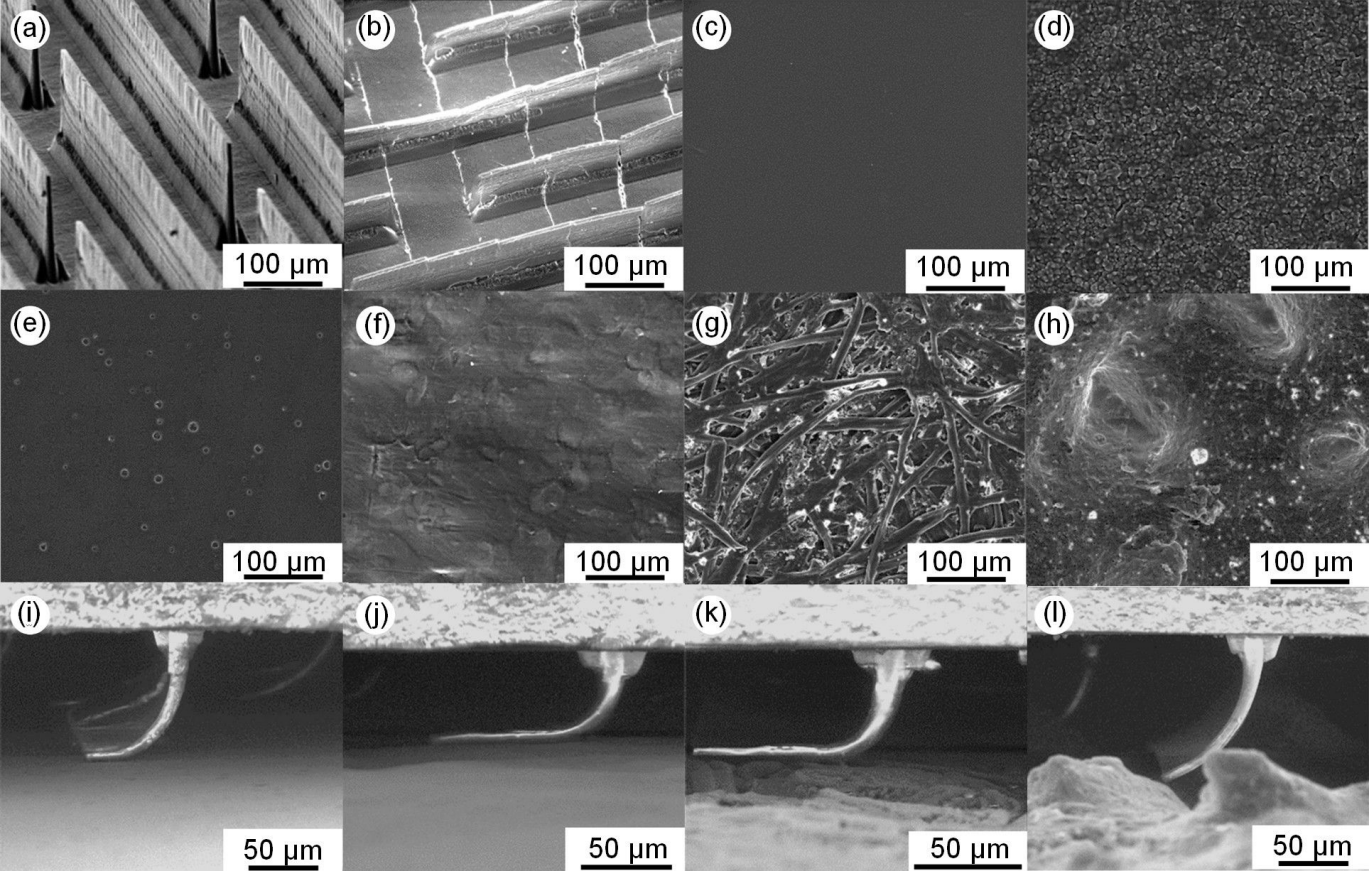
Characteristic images of tested polyvinylsiloxane (PVS) adhesive microstructures in (a) as-cast original and (b) split at 100 μm intervals shapes, the epoxy replicas of (c) glass slide, (d) 3 μm FibrMet disc, (e) refrigerator, (f) table desktop, (g) print paper, and (h) P150 abrasive paper used as counterface surfaces, and the wall-shaped microstructures in contact with the surfaces replicating the topography of (i) refrigerator, (j) table desktop #1, (k) print paper, and (l) P150 abrasive paper.

**Figure 2:**
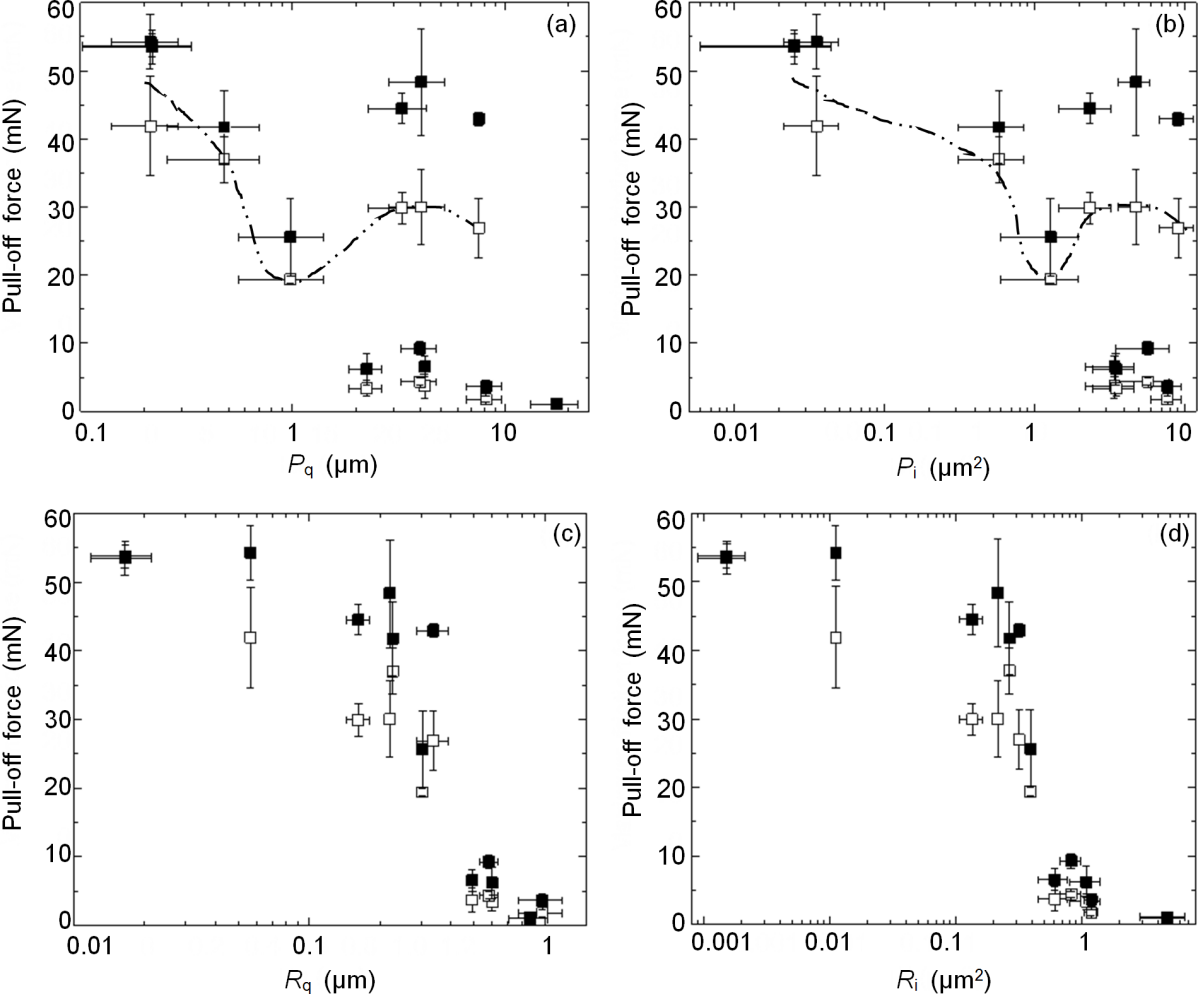
Pull-off force measured after preload of 20 mN on different rough surfaces and represented as a function of (a) *P*_q_ and (b) *P*_i_ calculated based on primary (unprocessed) counterface profiles, and (c) *R*_q_ and (d) *R*_i_ calculated based on roughness profiles obtained after Gaussian high-pass filtering with the cut-off wavelength of 10 μm. Open and filled markers represent mean values measured with original and split microstructures, respectively. Error bars represent the standard deviation. Dotted lines represent hand-drawn fits of the highest pull-off force values measured with the original microstructure over the whole range of profiles.

Pull-off forces measured with original and split wall-shaped adhesive microstructures against different rough surfaces (Figure 1, see Experimental for details) are shown in Figure 2. The surfaces are represented using the traditional root-mean-square deviation, and a new adhesion-oriented integrative characteristic [26] developed recently based on the Greenwood–Williamson approach [37]. The surface density of asperities, η, the mean radius of asperity summits, β, and the standard deviation of asperity height distribution, σ_s_, which are needed for calculation of the new integrative characteristic (σ_s_/βη) were obtained by analyzing asperity peaks identified in a 3D surface profile with a deterministic method based on eight nearest neighboring points [38].

Studying the relationship between the pull-off force and either the height (Figure 2a) or the hybrid height spacing (Figure 2b) parameters of the primary (as measured) profiles (root-mean-square deviation (*P*_q_) and new integrative characteristic (*P*_i_ = σ_s_/βη), respectively, Table 1), we do not see much correlation. This is because the measured primary profile characteristics are dominated by the low-frequency, long-wavelength components, known as waviness. The high-frequency, short-wavelength components, known as roughness, do not contribute much. To this end, wavy and smooth profiles may have approximately the same geometric characteristics as wavy and rough profiles, while their adhesive properties may differ greatly. This effect is evident in Figure 2a,b, where several surfaces having similar profile characteristics demonstrate very different pull-off forces. The lack of correlation between waviness-dominated characteristics and adhesion suggests that filtering the waviness out may result in a better correlation between the adhesive and geometrical properties of the studied counter surfaces.

**Table 1:** Mean surface profile characteristics obtained at five different locations on epoxy replicas of different objects.

	Table desktop #1	12 μm FibrMet disc	3 μm FibrMet disc	1 μm FibrMet disc	0.3 μm FibrMet disc	Print paper	Refrigerator	Wood block	Table desktop #2	Sputter coater	P150 abrasive paper	Glass slide

*P*_q_ (μm)	3.288	4.207	2.239	0.981	0.478	4.004	0.215	8.161	7.545	4.043	17.628	0.220
*P*_i_ (μm^2^)	2.344	3.417	3.505	1.282	0.580	5.696	0.036	7.726	9.072	4.744	49.033	0.025
*R*_q_ (μm)	0.162	0.493	0.600	0.304	0.226	0.581	0.057	0.977	0.338	0.220	0.867	0.017
*R*_i_ (μm^2^)	0.135	0.608	1.085	0.389	0.266	0.818	0.011	1.188	0.318	0.214	4.720	0.002

Looking at the highest pull-off forces measured over the whole range of profiles in Figure 2a,b, we can recognize the curves (dotted lines) associated with the idea of “critical roughness”, which was used to explain the reduction in the animal’s ability to attach to certain surfaces [18,39–40]. However, in light of the discussed lack of correlation between adhesion and waviness, and given that among the studied substrates we employed those used in [39–40], it may also be instructional to see how filtering out the waviness would reflect on the critical roughness.

The important question is how to find the division between roughness and waviness. Because the definition of the critical point at which roughness becomes waviness depends on the application (for instance, waviness on an optical lens may be considered as roughness on an automotive part), we have to account for the system’s performance with respect to its characteristic size. Given that the adhesive performance is determined by the system’s ability to form a large contact area while storing little elastic energy, we can define the above critical point as the point at which the adhesive flap bending needed for adaptation to surface irregularities becomes too energy consuming. To this end, the bending stiffness of the adhesive flaps is the decisive property and, hence, the flap thickness is the most important characteristic size (it is raised to the power of three in determination of the area moment of inertia for the flap cross-section). Assuming that flaps of 5 µm in thickness are not able to adapt to surface irregularities having a wavelength of less than 10 µm, we used this latter value as a cut-off length to filter out the waviness information from the primary profiles with the Gaussian high-pass filter [41].

The pull-off force, represented as a function of the parameters calculated based on the filtered roughness profiles, is shown in Figure 2c,d (root-mean-square deviation (*R*_q_) and new integrative characteristic (*R*_i_ = σ_s_/βη), respectively, Table 1). In line with the performance of thin-film-covered surface architectures [42–43], we can now see a clear negative correlation between the pull-off force and the roughness, with the integrative roughness *R*_i_ having better resolving power (4 vs 2 orders of magnitude in range) and higher Spearman’s rank correlation coefficient (−0.97 vs −0.95) than the root-mean-square roughness *R*_q_. This correlation supports our analysis of the relationships between adhesion, roughness and waviness, and proves that the adhesive performance of the wall-shaped microstructure depends on the microscale roughness of the counterface, most likely because it can adapt to a wavy but not to a rough surface. This finding implies that the profile measurements have to be properly conditioned to represent the adhesion data correctly.

It is also evident that, after filtering the waviness out, the idea of “critical roughness” does not work anymore, as we do not see a single most problematic roughness. Instead, the adhesive performance gradually degrades with increasing roughness, showing a range of roughness values that can be termed antiadhesive for the studied adhesive microstructures.

**Figure 3:**
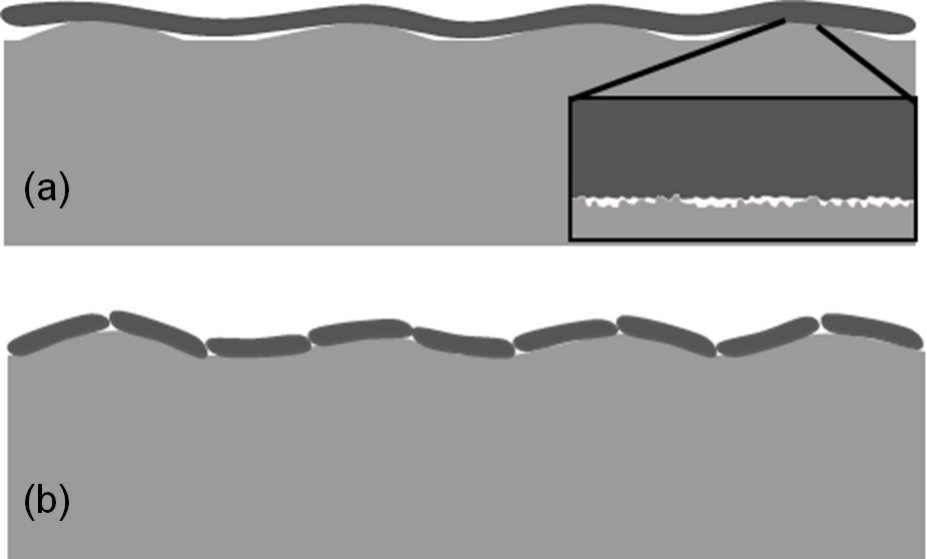
Schematic of the terminal parts of an (a) original as-cast and (b) split at 100 µm intervals wall-shaped microstructure in contact with a counter surface.

Analyzing the effect of contact splitting, we can undoubtedly see that, in agreement with common belief, it can effectively mitigate the roughness-driven reduction of the pull-off force. This observation is supported by a paired *t*-test (one-tailed *p*-value = 0.00152), according to which the mean pull-off force measured with the split microstructure exceeds that measured with the original microstructure by an amount that is greater than would be expected by chance. This key finding is demonstrated best in Figure 2c,d, where the roughness increase leads to a less pronounced reduction of the pull-off force if the microstructured surface is split, with this effect being more notable at the intermediate roughness, and vanishingly small at the very smooth and very rough surfaces. We can associate this effect with the changes in the real contact area. If the counterface is smooth, splitting the microstructure does not affect the real contact area. If the counterface is highly uneven, the real contact area is so small that its increase due to the better adaptability of the split microstructure (to wavy surfaces) cannot lead to that the increase in the pull-off force exceeds the measurement error. On the other hand, the improved adaptability of the split microstructure to wavy surfaces allows it to form larger contact area (Figure 3) on the surfaces with intermediate roughness, thus leading to a better attachment.

In addition to its better ability to adapt to uneven surface topography, a split microstructure may also demonstrate another effect that facilitates attachment. This effect is based on a nonlinear relationship between the peeling force and the peeling angle, as follows from the Kendall model of thin-film peeling [44]. It is evident that the Kendall model cannot be directly applied to interpret our results due to different boundary conditions. However, it provides a useful example that qualitatively illustrates the effect we may expect to see.

**Figure 4:**
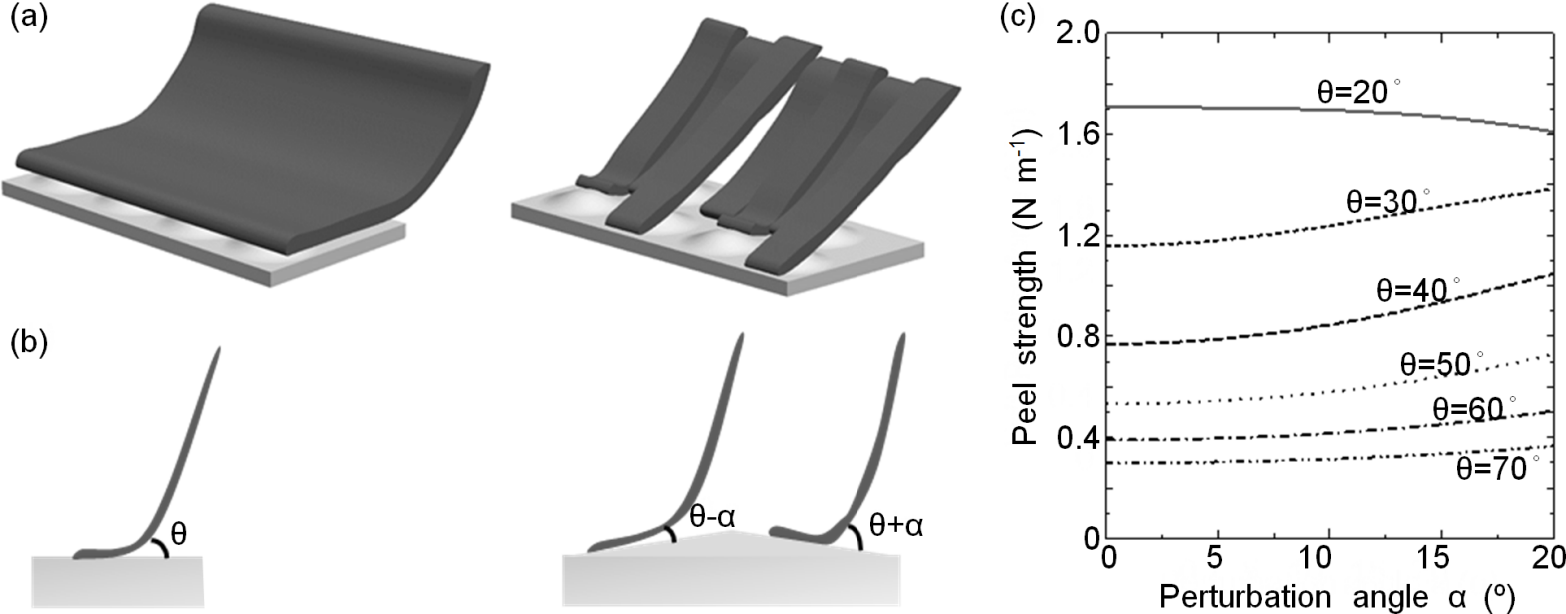
The effect of flap splitting on the contact geometry in (a) 3D representation and (b) 2D representation. (c) Peel strength of a split adhesive flap as a function of the peeling angle θ and the perturbation angle α.

Shearing an original and a split flap against an uneven substrate, we may expect that they will form contacts similar to those shown in Figure 4a, with the original flap being peeled at the same angle along all its width and the split (independent) flap being peeled at slightly different angles associated with the local surface slopes. Now, we can simplify and transpose this 3D model into a 2D space, as shown in Figure 4b, so the original flap peels at angle θ, while the two statistically equal fractions of the split flap peel at angles θ − α and θ + α, respectively, with α being a small perturbation angle defined by the surface topography. In this case, solving the Kendall equation ((2) in [44]) yields

[1] $$\frac{F}{b}=\text{d}E\left\{\left(\cos\theta \cos\alpha -1\right)+\frac{1}{2}\left[\begin{array}{l} \sqrt{{\left(\cos\left(\theta +\alpha \right)-1\right)}^{2}+\frac{2R}{\text{d}E}}\\ +\sqrt{{\left(\cos\left(\theta -\alpha \right)-1\right)}^{2}+\frac{2R}{\text{d}E}} \end{array}\right]\right\}$$

where *F* is the peeling force, *b* is the film width, *d* is the film thickness, *E* is the Young’s modulus, and *R* is the fracture energy.

Plotting the peel strength, which is the peeling force normalized by the film (flap) width, gives the curves shown in Figure 4c when the film (flap) thickness is 5 µm, the Young’s modulus is 3 MPa, the fracture energy is 0.2 N m^−1^, the peeling angle θ ranges from 20 to 70° and the perturbation angle α ranges from 0 to 20°. Studying these curves, we see that the peel strength can either increase or decrease with increasing perturbation angle α at different peeling angles θ. At peeling angles below ≈25°, the increase in perturbation angle (increase in surface unevenness) results in a decrease in the peel strength, while at peeling angles above ≈25°, the increase in perturbation angle results in an increase in the peel strength. Thus, given that the tested adhesive microstructure is loaded at high peeling angles in this work, we can conclude that splitting the adhesive microstructure in parallel to the peeling force may improve the attachment ability not only due to better adaptation to surface topography, but also due to the effective decrease of the peeling angle.

**Figure 5:**
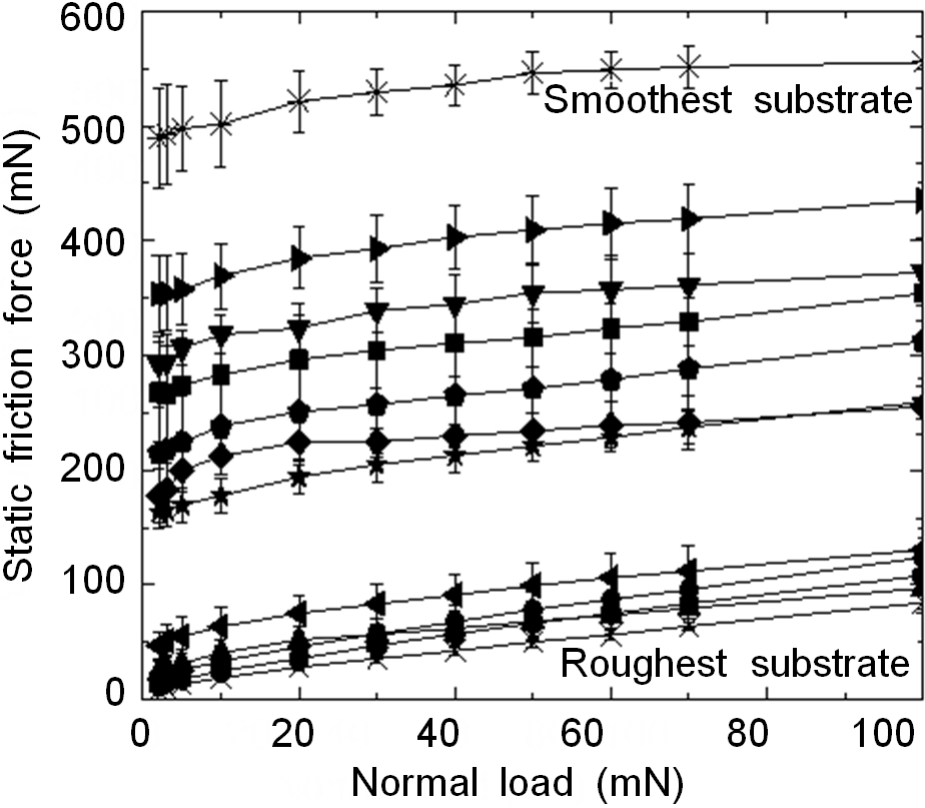
(a) Mean static friction force measured with original wall-shaped microstructures as a function of normal load on counter surfaces with different topography. Error bars represent standard deviation.

The friction force measured at the point of sliding inception on all substrates is presented in Figure 5 as a function of the normal load. Similar to our previous results [13], on a smooth counterface, we see extremely high friction starting from the very low normal loads (less than one hundredth of the measured friction) and growing with increasing load. In line with the performance of thin-film-covered surface architectures [43], deterioration of the surface finish results in the friction curves shifting towards lower values, while the effect of load is preserved. Obviously, this is explained by changes in the real contact area, which decreases with increasing roughness and increases with increasing load.

**Figure 6:**
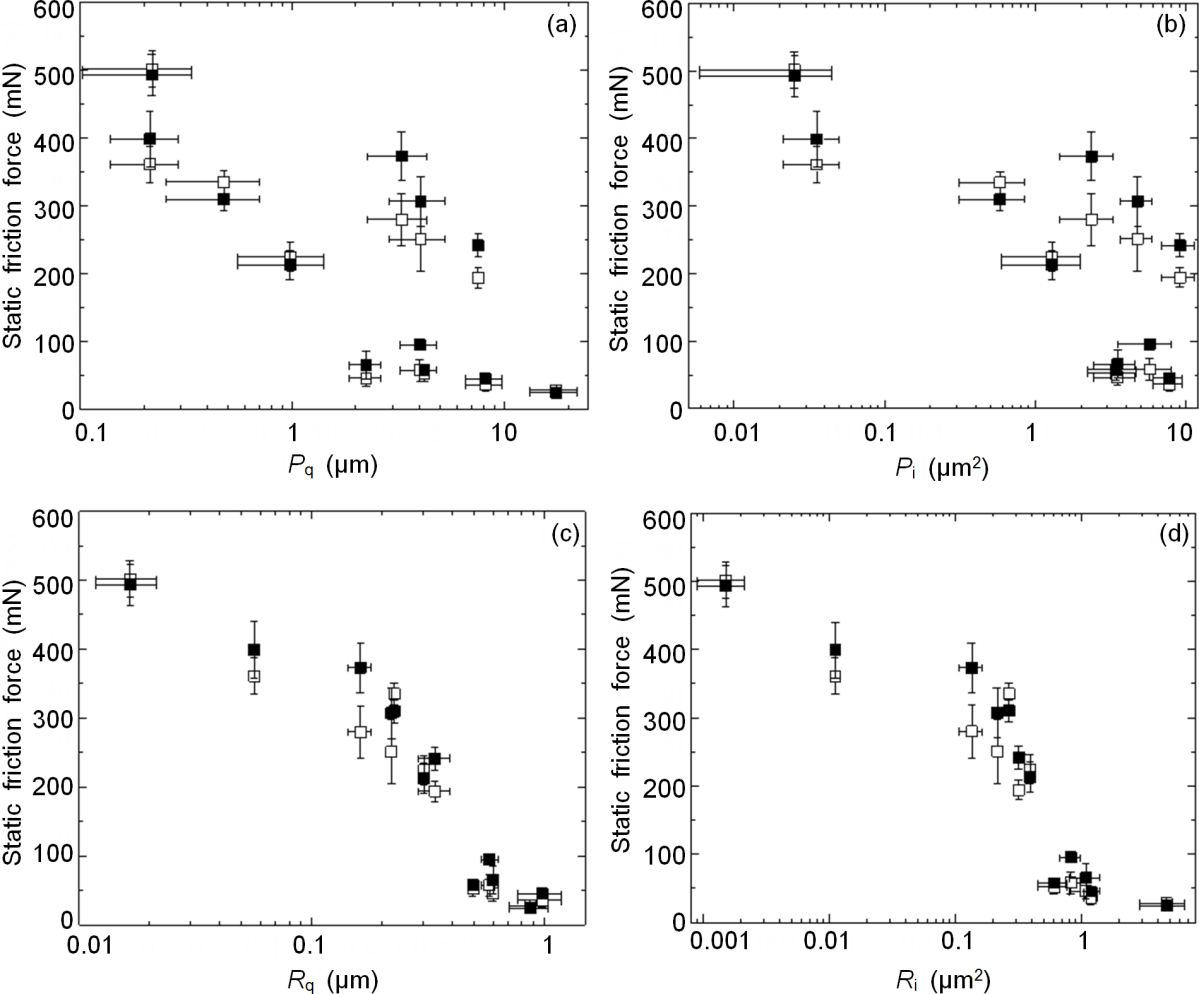
Static friction force measured under a normal load of 20 mN on different rough surfaces and represented as a function of (a) *P*_q_ and (b) *P*_i_ calculated based on primary (unprocessed) counterface profiles, and (c) *R*_q_ and (d) *R*_i_ calculated based on roughness profiles obtained after Gaussian high-pass filtering with the cut-off wavelength of 10 µm. Open and filled markers represent mean values measured with original and split microstructures, respectively. Error bars represent standard deviation.

Representing the friction data as a function of parameters characterizing surface topography yields the results shown in Figure 6. Here, in line with the data on pull-off force, we see that changes in friction correlate well with the changes in surface roughness, whereas having the data on waviness included in the analysis undermines the clarity of this effect. Splitting the wall-shaped microstructures also seems to increase the resistance to sliding, with this effect being more pronounced at intermediate roughness. Similarly to the pull-off force, this may happen due to a more efficient use of the available surface area by the split wall-shaped microstructures. On the very smooth and very rough substrates, this effect disappears because the real contact area is not affected by the contact splitting in the first case, and because the real contact area is so small in the second case that its growth due to the contact splitting is comparable to the measurement error. Interestingly, a paired *t*-test, according to which the mean friction force measured with the split microstructure exceeds that measured with the original microstructure by an amount that is greater than would be expected by chance, gives a one-tailed *p*-value of 0.0256. This figure is more than one order of magnitude larger than that obtained for the pull-off force, which suggests that the contact splitting may be more useful in mitigating the roughness-driven reduction of the pull-off force, while the force needed to start sliding is affected less.

## Conclusion

Based on the results presented in this work, we can conclude the following. First, because thin films can adapt to wavy but not to rough surfaces, the adhesion- and friction-driven attachment of the wall-shaped microstructure degrades, regardless of the surface waviness, when the surface roughness increases. Second, splitting the wall-shaped microstructure in parallel to the shear direction helps to mitigate the negative effect of the increasing surface unevenness by allowing the split microstructure to adapt more easily to the surface waviness as well as by allowing it to reduce the effective average peeling angle. These findings can guide the development of biomimetic shear-actuated adhesives that are suitable for operation not only on smooth but also on rough surfaces.

## Experimental

Microstructured surfaces with 140 µm high flaps (Figure 1a,b) were molded from PVS (Coltène Whaledent, Altstätten, Switzerland; Young’s modulus of about 3 MPa [45]) against a laser micro-machined grid (Oxford Lasers, Shirley, MA, USA) using a procedure described elsewhere [36]. Rectangular samples of 2.5 × 5 × 1 mm in size were cut out of the mold, so that the wall-shaped microstructures on each sample had a total peeling line length [34] of about 76 mm. To examine the effect of contact splitting, the wall-shaped microstructures were split at 100 μm intervals (Figure 1b) using a razor blade fixed to an LP150 low profile *X*–*Y* stage (Reliant Systems, Zimmerman, MN, USA).

Counterface samples of 20 × 5 × 1 mm in size were prepared from Spurr Epoxy resin EM0300 (Sigma-Aldrich, St. Louis, MO, USA) by replicating topography of twelve different surfaces (Table 1, Figure 1c–h) using a two-step molding method [46] and then cutting the samples to size. The surface topography was examined using a 3D optical profiler ContourGT-I (Bruker, San Jose, CA, USA). To obtain data on a large area without sacrificing resolution, all surface profiles were stitched from 24 regions of 1000 × 64 µm scanned at a magnification ×100 with a lateral sampling of about 0.1 µm.

The tests were carried out in a custom tribometer [47] able to measure pull-off and friction forces in ambient conditions as well as inside a Quanta 250 environmental SEM (FEI, Brno, Czech Republic). In this work, all force measurements were performed outside the SEM and the latter was used only to visualize different types of contact shown in Figure 1. Microstructured samples were mounted on the tribometer such that the wall-shaped microstructures were oriented perpendicular to the sliding/pulling direction and the following test sequence was run to measure the pull-off force. First, a counterface sample was moved perpendicular to the contact plane until the normal load of 20 mN was achieved. Then, the sample was moved parallel to the contact plane under the same normal load using a speed of 100 µm/s for the preliminary displacement of 300 µm needed for the flap alignment [36]. Next, the counterface sample was withdrawn from the contact at the speed of 100 µm/s at the pulling angle of 90° until it detached from the structured sample, and the detachment (pull-off) force was measured. The maximum friction force was measured at the instance of sliding inception while the counterface sample was slid against the microstructured sample under the constant normal load of 20 mN.

All samples were cleaned with common hand soap, deionized water and blown dry with nitrogen before use and they were inspected with an M125 optical stereomicroscope (Leica, Wetzlar, Germany). The SEM was operated in a low-vacuum mode (using water vapor) at 120 Pa and 10 kV to enable charge-free imaging of non-conductive PVS samples in their natural state. In order to image the epoxy samples in the SEM, they were coated with a 5 nm-thick-layer of Au/Pd using a Desk V sputter machine (Denton Vacuum, Moorestown, NJ, USA) operated for 180 seconds at 18 mA current and about 5 × 10^−2^ Pa Ar pressure. All tests were repeated at least four times. The temperature and relative humidity in the laboratory were 23–25 °C and 45–55%, respectively.
